# Green Surgery and Sustainability in Orthopaedic Operating Rooms: A Comprehensive Review of Environmental Initiatives

**DOI:** 10.7759/cureus.91369

**Published:** 2025-08-31

**Authors:** Ahmed Mohamed, Usman Fuad, Adham Elsayed, Alaa Elasad

**Affiliations:** 1 Trauma and Orthopaedics, Royal Cornwall Hospital, Truro, GBR; 2 General Practice, Zagazig University, Zagazig, EGY

**Keywords:** carbon footprint, circular economy, environmental impact, green operating room, operating room, orthopaedic surgery, sustainability, waste reduction

## Abstract

Healthcare systems worldwide contribute significantly to environmental degradation through substantial carbon emissions and waste generation. Operating rooms, particularly orthopaedic theatres, are among the most resource-intensive areas within healthcare facilities. With the growing recognition of climate change as a major health threat, sustainability initiatives in perioperative care have become increasingly important. This narrative review examines current sustainability initiatives in orthopaedic operating rooms, evaluating strategies to reduce waste generation and environmental impact while maintaining patient care. A comprehensive literature review was conducted, examining peer-reviewed articles, systematic reviews, and policy documents related to environmental sustainability in operating rooms, with a specific focus on orthopaedic surgery. Sources included waste audit studies, carbon footprint analyses, and reports on green operating room initiatives published between 2013 and 2024. Sustainability initiatives in orthopaedic operating rooms can significantly reduce energy consumption and waste production. However, successful implementation faces challenges. Collaboration between different healthcare departments and sustainability multi-disciplinary team discussions are recommended for overcoming future challenges.

## Introduction and background

Healthcare systems worldwide are monitoring their substantial environmental impact while maintaining efficiency in providing good standards of patient care. The healthcare sector accounts for approximately 4-5% of global carbon dioxide (CO₂) and greenhouse gas emissions. The National Health Service (NHS) is responsible for 25 megatons of CO₂ equivalents annually. Additionally, it generates over 500,000 tonnes of waste [1]. These gases contribute to global warming by trapping heat within the atmosphere, thereby leading to climate change [2].

Orthopaedic surgery presents unique environmental challenges compared to other surgical specialties due to its reliance on complex instrumentation, single-use implants, extensive packaging materials, and energy-intensive equipment [3]. Recent studies have demonstrated that major joint arthroplasty procedures generate between 7 kg and 16 kg of plastic waste per case, with total hip arthroplasty producing approximately 14.6 kg CO₂ equivalent emissions and total knee arthroplasty generating 15.8 kg CO₂ equivalent emissions per procedure [4,5].

The green surgery terminology has evolved to maintain reductions in energy consumption in the operating rooms, making surgery safer for patients and the planet. The concept of sustainable healthcare has evolved from mounting evidence of the impact of climate change on human health [6,7]. The World Health Organization (WHO) has predicted an additional 250,000 deaths annually between 2030 and 2050 due to climate change effects, with air pollution contributing to over eight million deaths yearly [8]. Healthcare systems have prompted leaders to recognize environmental sustainability as a clinical and ethical imperative [3].

The NHS recognizes its environmental responsibilities through its commitment to achieving net-zero carbon emissions by 2045 [8]. This ambitious target requires collaboration between all healthcare delivery areas, particularly operating rooms, considering their disproportionate environmental impact relative to their size.

Understanding the environmental impact of orthopaedic surgery requires examination of the entire care pathway that goes beyond operating room waste to include supply chain emissions, sterilization processes, postoperative care requirements, waste disposal, and treatment of complications [9,10]. This comprehensive perspective is essential for developing effective sustainability strategies that address the root causes of environmental impact rather than merely the symptoms. In this review, we discuss the environmental impact of orthopaedic operating rooms on climate change, different ways of maintaining sustainability, and future challenges and directions that should be followed to reduce this environmental impact.

## Review

Methods

A comprehensive narrative review was conducted to examine the sustainability initiatives in orthopaedic operating rooms. A literature search was performed using multiple electronic databases, including PubMed, MEDLINE, Embase, and Cochrane Library. Search terms included combinations of "sustainability", "orthopaedic surgery", "operating room", "environmental impact", "waste reduction", "carbon footprint", and "green surgery".

The search was limited to peer-reviewed articles, systematic reviews, policy documents, and organizational reports published in English between 2013 and 2024. Grey literature from professional organizations, including the British Orthopaedic Association, Royal College of Surgeons, and NHS sustainability initiatives, was also included. Studies focusing on environmental sustainability in surgical settings, particularly in orthopaedic surgery, were prioritized.

Data extraction focused on waste generation patterns, carbon footprint measurements, energy consumption, sustainability interventions, implementation challenges, and economic considerations. Quality assessment was performed for the included studies, with preference given to systematic reviews, large cohort studies, and institutional reports with robust methodologies. The 5Rs framework (reduce, reuse, recycle, rethink, and research) was used as a conceptual model to organize the findings and recommendations.

Current environmental impact and scale of the challenge

Operating rooms are particularly carbon-intensive environments, consuming approximately three to six times more energy than other departments and producing significant amounts of waste for their size [2]. One operating theatre in a UK teaching hospital produced a comparable annual carbon footprint to over 2,000 homes [11]. The major sources of carbon emissions in orthopaedic operating rooms are detailed in Table 1, which shows that anaesthetic gases contribute the largest proportion of emissions, accounting for 42-88% of theatre emissions.

**Table 1:** Common Carbon Emissions in Orthopaedic Operating Rooms HVAC: Heating, Ventilation, and Air Conditioning; LED: Light-Emitting Diode

Emission Category	Contribution to Total Footprint	Specific Sources	CO₂ Equivalent Impact	Reference
Anaesthetic Gases	42-88% of theatre emissions	Nitrous oxide (90% of anaesthetic emissions), desflurane, sevoflurane, isoflurane	Desflurane: 20x greater than sevoflurane	[12,13]
Energy Consumption	58% of non-anaesthetic emissions	HVAC systems (60-70% of theatre energy), LED vs traditional lighting, medical equipment	20-30% reduction possible with occupancy-based ventilation	[14,15]
Medical Devices & Instruments	32% of the remaining footprint	Single-use instruments, implant manufacturing, packaging materials	30-50% energy savings with reusables	[16,17]
Transportation & Logistics	5-10% of total emissions	Implant delivery, patient transport, staff commuting	159.6 kg CO₂ per 100 prostheses (packaging alone)	[5]
Waste Management	3-5% of total emissions	Incineration of clinical waste, inappropriate segregation	50x cost increase with poor segregation	[18]
Water Consumption	1-3% of total emissions	Surgical scrubbing, equipment cleaning, sterilization	2.7 million litres annual savings potential	[19]

Table 2 demonstrates the significant variation in waste generation across different orthopaedic procedures, with revision arthroplasty producing the highest amounts of waste and carbon emissions, while arthroscopic procedures generate the least waste.

**Table 2:** Common Orthopaedic Procedures and Waste Generation Waste generation varies significantly based on procedural complexity, institutional practices, and surgical approaches. These ranges reflect the variations across different studies and healthcare systems. ORIF: Open Reduction Internal Fixation

Procedure Type	Total Waste per Case (kg)	Plastic Waste (kg)	Recyclable Waste (%)	CO₂ Equivalent Emissions (kg)	Reference
Total Hip Arthroplasty	12.5-16.5	7.28	43.9%	14.6	[4,5,9,20]
Total Knee Arthroplasty	11.8-15.2	7.63	43.9%	15.8	[4,5,9,20]
Hip Hemiarthroplasty	8.5-12.0	5.2-6.8	38.5%	10.2-12.4	[9,20]
Arthroscopic Procedures	3.2-5.8	2.1-3.4	52.3%	4.8-7.2	[9,20]
Fracture Fixation (ORIF)	6.8-9.5	4.2-5.9	41.2%	8.5-11.3	[9,20]
Spinal Fusion	15.2-22.1	9.8-14.2	35.7%	18.9-25.6	[9,20]
External Fixation	4.1-6.7	2.8-4.1	47.8%	5.9-8.4	[9,20]
Revision Arthroplasty	18.7-25.3	12.1-16.8	31.4%	22.8-31.2	[4,9,20]

The evolution of orthopaedic surgery toward increasingly complex procedures and sophisticated technology has increased the environmental footprint [20]. The transition from reusable to single-use instrumentation, driven by infection control concerns, has substantially increased waste generation [21,22]. Current data reveal that total hip arthroplasty procedures produce a mean plastic waste of 7.28 kg, whereas total knee arthroplasty generates 7.63 kg of plastic waste per case [9]. When considering broader waste categories, elective procedures can generate up to 16.5 kg of total waste per case [5].

The carbon footprint of orthopaedic procedures extends beyond immediate operational emissions. Supply chains, including the manufacturing, packaging, and transportation of implants and instruments, significantly contribute to the overall environmental burden [23]. Healthcare waste disposal is another challenge that costs between $760 and $935 billion annually in the United States alone [24]. This economic burden reflects not only immediate costs but also the broader societal impact of environmental degradation.

Anaesthetic considerations and gas management

Anaesthetic gases are potent greenhouse gases and are released directly into the atmosphere after use. Therefore, their impact on global warming is significant. They contribute up to 88% of the carbon footprint of operating theatres [25]. Volatile anaesthetic gases account for approximately 5% of the NHS carbon footprint. Nitrous oxide alone is responsible for 90% of the anaesthetic-related emissions [26]. The environmental impact varies dramatically between different anaesthetic agents, with desflurane having a carbon footprint 20 times greater than that of sevoflurane [26,27]. Both agents are used for induction and maintenance of anaesthesia.

Regional and local anaesthesia techniques produce substantially lower carbon emissions than volatile agents [28]. For orthopaedic procedures, using regional techniques when clinically appropriate reduces the carbon footprint immediately without compromising patient care.

Pain management strategies also present opportunities for sustainable practices. Traditional nitrous oxide use for fracture reduction and minor orthopaedic procedures can be replaced with methoxyflurane (Penthrox), which has a significantly lower environmental impact [29]. The development of anaesthetic gas calculators enables clinicians to make informed choices based on environmental impact while maintaining clinical effectiveness [30].

The comprehensive 5Rs framework for sustainable practice

To directly address the substantial waste and carbon emissions identified, the "5Rs" framework offers a comprehensive strategy. This approach not only aims to mitigate environmental impact but also aligns with the need for sustainable transformation in orthopaedic surgery practices [31]. This model has been enhanced by some institutions with a "6th R" - repurposing - to address clean, unused surgical supplies that retain value for community organizations and educational purposes [32]. Each component offers specific opportunities for environmental improvement while maintaining patient safety.

Reduce: Minimizing Resource Consumption

The reduction strategy is the most effective method for optimizing energy consumption. Waste reduction can be achieved through the application of educational programmes and the establishment of protocols for conservative energy consumption [33]. Prevention is always better than treatment. Osteoporosis treatment could prevent up to 25% of hip fractures [34]. Implementing public health policies that focus on bone health, injury prevention, and early intervention can significantly reduce the overall demand for orthopaedic surgery.

The operating room energy consumption patterns are a significant challenge. Heating, air conditioning, and ventilation systems use approximately 60-70% of the total operating room energy [35]. This can be reduced by 20-30% if traditional systems are replaced with occupancy-based control systems that provide the same air quality standards [36]. Lighting optimization through LED replacement programs can reduce energy consumption by 50-80% while improving light quality and service life and reducing heat production [19].

Surgical tray optimization represents an immediate opportunity for waste reduction in operating rooms. Studies have demonstrated that custom surgical packs designed for specific procedures can reduce waste by 40% compared with standard configurations [37]. This requires effective communication between surgeons, nurses, and suppliers to customize the surgical sites to reflect real rather than anticipated needs.

Efforts are ongoing to replace disposable products with reusable alternatives where clinically appropriate. Each substitution requires careful risk-benefit analysis to ensure patient safety while achieving environmental benefits.

Reuse: Extending Product Lifecycles

Reusable textiles, including surgical gowns and drapes, reduce energy consumption by approximately 30-50% compared to their disposable alternatives while maintaining equivalent cost, comfort, and infection prevention effectiveness [38]. External fixation component recycling is a promising approach for improving sustainability. Recycled external fixators are safe, cost-effective, and well-received by patients; however, recycling remains uncommon because of logistical challenges [39]. A national recycling protocol would streamline these processes and reduce the energy and resources required to manufacture new components.

Walking aids and mobility equipment represent underutilized opportunities for reuse. Only 21% of crutches provided by fracture clinics and emergency departments are returned for reuse [40]. Successful initiatives, such as "crutch amnesty" programmes, have demonstrated improved return rates and should be implemented more widely [41].

Recycle: Material Recovery and Processing

Approximately 99% of non-contaminated waste from primary joint arthroplasty is recyclable. However, almost none of these materials are recycled; instead, they are either incinerated or sent to landfills [4]. This is primarily due to inadequate waste classification protocols and facilities in operating theatres, which increase disposal costs by up to 50-fold [42].

Blue wraps are used in large quantities in orthopaedic surgery to keep sets and implants sterile. Diverting blue wraps from the clinical waste stream to the recycling stream offers immediate benefits to hospitals. These polypropylene materials can be recycled into plastic products, with pilot programmes demonstrating potential revenue generation and annual savings exceeding $174,000 for large institutions [43].

Medical device remanufacturing represents an emerging opportunity for high-value orthopaedic equipment. The concept of a circular economy (CE) has emerged from the need to recycle products and stop the single-cycle concept of production and disposal. The value of products and the materials they are made of can be maintained in the economic cycle by recycling the products [44]. Metal recycling programmes can recover valuable materials from orthopaedic implants and instruments. Stainless steel, titanium, and other alloys used in orthopaedic surgery have significant recycling value and can be processed into high-quality materials for various applications [45].

Maintaining good standards of sustainable practice requires the implementation of national policies, dedicated educational programmes, mandatory training on waste segregation, and the provision of recycling facilities in operating theatres.

Rethink: Challenging Traditional Approaches

Rethinking what we are doing and how to improve it is a key step in improving adherence to sustainability. Procedure location optimization involves reconsidering the locations where orthopaedic procedures are performed. Specific cases that require low resources can be moved from the main theatres to smaller minor theatres to reduce energy consumption and resource utilization while maintaining the quality of care. Day-case surgery programs can eliminate overnight stays and associated resource consumption [46].

Minimally invasive surgical techniques should be considered where indicated, as they require smaller surgical packs, generate less waste, and may reduce energy consumption compared with traditional open procedures [47]. Rethinking also includes considering other treatment options that can be offered to patients. Nonoperative management strategies, when clinically appropriate, can completely avoid the environmental impact of surgery. Enhanced recovery programs can reduce the length of stay and associated resource consumption while improving patient outcomes [48].

Research: Evidence-Based Improvement

Assessing the entire product life cycle provides a comprehensive evaluation of the environmental impacts of each stage. This helps identify areas for improvement in compliance with sustainability measures throughout the supply chain [49].

The research role in sustainability has evolved as companies have been reviewing their business models and environmental impact, as all investors prefer clean and economically sustainable industries. This includes materials science research on biodegradable implants, energy-efficient equipment development, and process optimization studies [50].

Continuous improvement programmes use systematic data collection and analysis to identify opportunities for environmental improvements. Regular auditing, performance monitoring, and feedback systems enable the iterative enhancement of sustainability practices while maintaining quality [51].

Repurposing: Make the Best Use of Our Resources

Unused but clean surgical materials from operating rooms can be sustainably repurposed through structured donation programmes and internal-reuse systems. Items such as gloves, sutures, and drapes can support under-resourced healthcare settings, disaster relief efforts, and local medical training programs. Hospitals may also redirect supplies for simulation or veterinary use if infection control protocols are followed. Partnering with nonprofit organizations helps ensure safe and ethical redistribution, whereas internal systems facilitate nonclinical reuse. Such practices not only reduce medical waste but also promote environmental and social responsibility in healthcare.

Challenges and barriers to implementation

Despite strong evidence of sustainability benefits, significant barriers continue to prevent the widespread implementation of these systems. Urgent regulatory requirements and infection control protocols often prioritize safety over environmental considerations, creating a tension between sustainability goals and clinical obligations [52]. Time and resource modifications required to implement new changes are other barriers. Patients' expectations and demands for quick and efficient healthcare services are increasing. This challenge inhibits the desire to adopt new sustainable protocols that may compromise established practices [53,54]. Cost considerations are also important in this context. Healthcare systems prefer immediate financial returns to long-term savings [55]. Organizations operate under tight financial constraints that prioritize immediate needs over long-term environmental benefits [56].

Cultural resistance and change management challenges can impede implementation, even when financial and technical barriers are addressed [57]. Healthcare professionals may resist process changes that disrupt established routines or require additional training because of time constraints. Hierarchical organizational structures and professional autonomy can complicate the implementation of standardized sustainability protocols [58].

Technical challenges include the limitations of alternatives to current practices and products. Some sustainability initiatives may not be feasible because of clinical requirements, regulatory constraints or technological limitations [59]. Certain single-use medical devices may lack clinically acceptable reusable alternatives, thereby limiting waste reduction [22]. Some sustainability suggestions, such as using new devices in operating rooms, may be very expensive, especially in developing countries [60].

Economic impact

Studies and reviews have reported that implementing sustainable practices, such as reducing the use of single-use instruments, optimizing surgical packs, and switching to reusable drapes and equipment, can yield major cost reductions. The "lean and green" model in hand surgery, which aims to streamline instrument trays and use smaller drapes, was found to be approximately two-thirds cheaper per case than traditional methods. This technique reduces the carbon footprint of carpal tunnel release by 80% [61]. Minimizing single-use items and customizing surgical packs led to reductions of 2.8 tonnes of waste and over $13,000 in supply costs across 1,099 hand procedures, with per-case savings of 13% and 55% in waste and material costs, respectively [62].

The economic benefits of various sustainability initiatives are summarized in Table 3, which demonstrates the financial viability of most interventions, with payback periods ranging from two to four months for walking aid reuse programs to 18-24 months for occupancy-based HVAC (heating, ventilation, and air conditioning) controls.

**Table 3:** Economic Impact of Sustainability Initiatives in Orthopaedic Surgery HVAC: Heating, Ventilation, and Air Conditioning; LED: Light-Emitting Diode

Sustainability Initiative	Initial Investment	Annual Savings	Payback Period	Additional Benefits	Reference
LED Lighting Replacement	£15,000-25,000 per theatre	£8,000-12,000 per theatre	2-3 years	50-80% energy reduction, improved lighting quality	[19]
Blue Wrap Recycling Program	£5,000-10,000 setup	£174,000+ for large institutions	6-12 months	Revenue generation, waste diversion	[43]
Custom Surgical Pack Optimization	£2,000-5,000 implementation	£13,000 per 1,099 procedures	3-6 months	40% waste reduction, 55% material cost savings	[37,62]
Reusable Textiles (Gowns/Drapes)	£20,000-40,000 initial	£15,000-30,000 per year	12-18 months	30-50% energy savings, infection control maintained	[38]
Occupancy-Based HVAC Controls	£30,000-50,000 per theatre	£25,000-40,000 per theatre	18-24 months	20-30% energy reduction, maintained air quality	[35,36]
Walking Aid Reuse Program	£1,000-3,000 setup	£8,000-15,000 per year	2-4 months	Equipment cost avoidance, community benefit	[40,41]
Lean and Green Hand Surgery Model	£5,000-8,000 training	£500-800 per case	6-10 cases	80% carbon footprint reduction, 67% cost reduction	[61]
Regional Anaesthesia Protocols	£10,000-15,000 training	£200-400 per case	25-75 cases	Up to 88% anaesthetic emissions reduction	[28,30]

Future directions and emerging opportunities

The future of sustainability in orthopaedic operating rooms requires consideration of environmental impact in all aspects of care delivery [63]. The application of modern technology and artificial intelligence (AI) will enable the tracing of medical equipment and implants and facilitate automatic requests from the supply chain. This will reduce carbon emissions from frequent, unnecessary material deliveries to hospitals [64].

The application of CE principles will help both supply chains and healthcare systems maximize resource utilization and minimize waste generation [65]. In orthopaedic surgery, applications might include the remanufacturing of medical devices, material recovery from surgical waste, and closed-loop supply chains. Innovations in materials science create opportunities for the development of sustainable medical devices. Biodegradable materials, recyclable plastics, and bio-based alternatives can reduce environmental impact while maintaining clinical efficacy [66].

More importantly, professional education and training programs represent a quick, cheap, and effective way to maintain sustainability adherence. Future healthcare professionals must understand environmental impact assessment, sustainable practice design, and change management techniques. Mandatory training on waste disposal and ways to save energy, such as turning off lights when not needed and using water sparingly when getting scrubbed, is important for achieving immediate results.

National and international guidelines are needed to control anaesthetic emissions, including clear protocols for using local and regional anaesthesia when applicable instead of general anaesthesia and sedation. Applying these measures will have a significant impact on reducing greenhouse gas emissions while maintaining the quality of care.

## Conclusions

Sustainability initiatives in orthopaedic operating rooms reduce environmental impact while generating cost savings and improving efficiency. However, successful implementation requires systematic approaches that address technical, cultural, and financial challenges while maintaining an unwavering commitment to patient safety and clinical efficacy. The 5Rs framework provides practical guidance for developing comprehensive sustainability programs that address multiple aspects of operating room sustainability. Prevention through public health initiatives offers the greatest environmental benefits, whereas waste reduction and proper waste segregation provide immediate opportunities for improvement. Energy conservation and supply chain optimization can generate substantial long-term savings through systematic implementation, which is supported by strong leadership and comprehensive education.

The unique characteristics of the orthopaedic surgery position this specialty to lead healthcare sustainability efforts. The substantial resource requirements and environmental impact of orthopaedic procedures create significant opportunities for meaningful improvement. The challenge ahead involves scaling successful initiatives across healthcare systems while adapting to local contexts and constraints. This will require continued innovation, policy support, and cultural change that positions sustainability as a core component of healthcare excellence rather than an optional consideration. The orthopaedic community can demonstrate leadership in this critical area while contributing to broader healthcare transformation toward environmental sustainability.
